# Smoking cessation after diagnosis of COPD is associated with lower all-cause and cause-specific mortality: a nationwide population-based cohort study of South Korean men

**DOI:** 10.1186/s12890-023-02533-1

**Published:** 2023-07-03

**Authors:** Jang Ho Doo, Sung Min Kim, Young Jun Park, Kyae Hyung Kim, Yun Hwan Oh, Ji Soo Kim, Sang Min Park

**Affiliations:** 1 Seoul National University College of Medicine, Seoul, Republic of Korea; 2 Department of Biomedical Science, Seoul National University Graduate School, Seoul, Republic of Korea; 3 Medical Research Center, Genomic Medicine Institute, Seoul National University, Seoul, Republic of Korea; 4 Department of Family Medicine, Seoul National University Hospital, Seoul National University College of Medicine, Seoul, Republic of Korea; 5 Department of Family Medicine, Chung-Ang University Gwangmyeong Hospital, Chung-Ang University College of Medicine, Gwangmyeong-Si, Republic of Korea; 6 International Healthcare Center, Seoul National University Hospital, Seoul National University College of Medicine, Seoul, Republic of Korea; 7 Department of Family Medicine and Biomedical Sciences, Seoul National University College of Medicine, 101 Daehak-ro, Jongno-gu, Seoul, 03080 Republic of Korea

**Keywords:** Smoking cessation, Mortality, COPD, Newly diagnosed COPD, Quitting smoking

## Abstract

**Background:**

The most effective way to halt the advancement of COPD is smoking cessation. However, limited data are available on the question of whether quitting smoking within two years after COPD diagnosis reduces the risk of mortality. The goal of our research was to analyze the relationship between quitting smoking after COPD diagnosis and the risks of all-cause and cause-specific mortality, using the Korean National Health Insurance Service (NHIS) database.

**Methods:**

This study included 1,740 male COPD patients aged 40 years or more who had been newly diagnosed within the 2003–2014 time period and had smoked prior to their COPD diagnosis. The patients were categorized into two groups according to their smoking status after COPD diagnosis: (i) persistent smokers (ii) quitters (smoking cessation within two years of COPD diagnosis). Multivariate Cox proportional hazard regression was performed to determine the adjusted hazard ratio (HR) and 95% confidence interval (CI) for both all-cause and cause-specific mortality.

**Results:**

Among 1,740 patients (mean age, 64.6 years; mean follow-up duration, 7.6 years), 30.5% stopped smoking after COPD diagnosis. Quitters gained a 17% risk reduction in all-cause mortality (aHR, 0.83; 95% CI, 0.69–1.00) and a 44% risk reduction in cardiovascular mortality (aHR, 0.56; 95% CI, 0.33–0.95) compared with persistent smokers.

**Conclusion:**

Our study found that patients who quit smoking within two years after COPD diagnosis had lower risks of all-cause and cardiovascular mortality relative to persistent smokers. These results can be used to encourage newly diagnosed COPD patients to stop smoking.

**Supplementary Information:**

The online version contains supplementary material available at 10.1186/s12890-023-02533-1.

## Background

COPD is a respiratory disease caused by inhalation of harmful particles or gases that initiate abnormal inflammatory reactions in the lungs [1]. In 2020, the global prevalence of COPD among people between the ages of 30 and 79 years was 10.3%, among 391.9 million total sufferers [2]. Persistent smoking is the leading cause of COPD, and contributes to rapid decline of lung function [2, 3]. It has been suggested by various studies that quitting smoking is the most effective way to delay the advancement of COPD and improve impaired lung function [4]. Thus, the Global Initiative for Chronic Obstructive Lung Disease (GOLD) guideline instructs COPD patients to quit smoking using both behavioral therapy and pharmacological agents [5]. Individuals tend to follow this advice, in that that the likelihood of quitting smoking increases when diagnosed with a tobacco-related disease [6]. Similarly, after receiving information on COPD, 84.1% of high-risk smoking patients showed an increased willingness to quit smoking [7].

Despite numerous studies demonstrating the beneficial effect of smoking cessation on survival rates in individuals with COPD, accurate assessment of the long-term impact of smoking cessation on mortality has proved challenging, due to studies’ limited sample sizes and short follow-up periods [8–10]. Nonetheless, it has been convincingly shown that smoking cessation has significant long-term benefits in terms of reducing mortality in young patients with asymptomatic COPD [11]. However, since most COPD patients do not seek medical advice until their condition has progressed significantly [12], it is important to show that smoking cessation at COPD diagnosis leads to reduced risk of mortality. In the present study, we evaluated the implementation of smoking cessation by comparing patients’ smoking status before and after COPD diagnosis and analyzed the mortality risk reduction in those who had quit after COPD diagnosis. We aimed to examine the association of smoking cessation within two years of diagnosis with mortality compared to persistent smoking in newly diagnosed COPD male patients using the National Health Insurance Service (NHIS) database.

## Methods

### Data source

We obtained data from the Korean National Health Insurance Service-National Health Screening Cohort (NHIS-HEALS) database covering the period from January 2002 to December 2019. Since 1989, the NHIS has provided 97% of the Korean population with health coverage. Under the National Health Screening Program, it is recommended that all citizens 40 years old or older have a medical examination every two years. At that time, urine and blood tests, physical measurements, and a questionnaire about health behaviors and sociodemographic information are collected from participants and combined with hospital usage, death register information, and medication prescription data to create the NHIS-HEALS dataset using a random sampling method [13].

### Study population

The study participants were male patients aged ≥ 40 years who had been newly diagnosed with COPD between January 2003 and December 2014. These newly diagnosed COPD patients were defined using the ICD-10 codes for COPD (J42.x–J44.x (except J430)), and all had been prescribed one or more of the following medications at least twice per year: LAMA (long acting muscarinic antagonist), LABA (long acting beta-2 agonist), ICS (inhaled corticosteroid), ICS plus LABA, SAMA (short acting muscarinic antagonist), SABA (short acting beta-2 agonist), methylxanthine, systemic corticosteroid or systemic beta-2 agonist [14]. As 89% of female COPD patients were never smokers [15], we excluded them from study participants.

### Smoking status

Patients were divided into persistent smokers and quitters according to the pre-to-post-COPD-diagnosis continuation or change of smoking status, respectively. Patients’ smoking status was assessed using self-reported questionnaires completed during health examinations before and after COPD diagnosis, respectively. In each questionnaire, patients had to select one response among “never-smoker,” “former-smoker,” and “current smoker.” This study included only current smokers in the pre-diagnosis category so as to demonstrate the benefits of quitting smoking after COPD diagnosis. Among the pre-diagnosis current smokers, those who sustained their smoking habit after COPD diagnosis were classified as persistent smokers, and those reporting that they were former smokers after COPD diagnosis were classified as quitters.

### Determination of outcomes

**Fig. 1:**
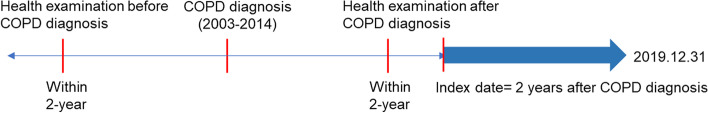
Study design

The study outcomes were all-cause and cause-specific mortality among patients who had quit smoking after COPD diagnosis compared with persistent smokers. Participants were selected based on the criterion of surviving a minimum of two years after their COPD diagnosis. The index date was two years after the COPD diagnosis. The cause of death was categorized using ICD-10 codes as cancer (C00-C97), cardiovascular disease (I00-I99) or respiratory disease (J00-J99), and more specifically, as lung cancer (C34), ischemic heart disease (I20-I25), stroke (I60-I69) or COPD (J42-J44). The follow-up period for all of the participants started at the index date and ended on the date of the participant's death or December 31st, 2019, whichever came first (Fig. 1).

### Covariates

We adjusted for confounding by including the following factors in our analysis: age, household income, systolic blood pressure, total cholesterol, fasting serum glucose, alcohol consumption, physical exercise, body mass index (BMI), Charlson comorbidity index, and severity of COPD. Each variable except Charlson comorbidity index had been obtained from the post-COPD-diagnosis health examination (i.e., the second health examination). Household income had been recorded as deciles on the basis of the NHIS premium, and for the purposes of the present study, was divided into quartile levels. The ‘severe COPD’ group consisted of patients who had visited a tertiary hospital and been prescribed combinations of ICS + LABA + LAMA, ICS + LABA + systemic corticosteroid, or LAMA + systemic corticosteroid on more than one occasion. The remaining study subjects were classified as the "not severe" group [16]. The Charlson comorbidity index was calculated according to the ICD diagnostic codes recorded between 1 January, 2002 and the index date using the same algorithms as those reported in a previous study [17]

### Statistical analysis

Baseline characteristics were presented as means with standard deviations for continuous variables and as numbers with percentages for categorical variables. We performed multivariate Cox proportional hazard regression to calculate the adjusted hazard ratio (HR) and 95% confidence interval (CI) for both all-cause mortality and cause-specific mortality based on smoking status after COPD diagnosis. Persistent smokers were the reference group, and so it was possible to evaluate the HR of quitters relative to persistent smokers. In this study, data were compiled and the statistical analysis was performed with SAS version 9.4 (SAS Institute, Cary, NC, USA). The level of significance was a *p*-value below 0.05, which was determined using a two-sided approach.

In a supplementary analysis, population attributable fraction (PAF) and numbers needed to treat (NNT) were calculated using the adjusted HR by the method suggested in previous studies [18, 19].

A subgroup analysis was performed for all-cause mortality risk according to smoking status as stratified by age group (< 60 years, ≥ 60 years), alcohol consumption (No, Yes), Charlson comorbidity index (CCI score < 3, CCI score ≥ 3), severity of COPD (not severe, severe) and presence of hypertension, cancer or cardiovascular disease (yes, no for each). Presence of comorbidities was identified by analysis of information on inpatient and outpatient visits and prescription records for the period between 1 January, 2002 and the index date. A *p* score for interaction < 0.1 was regarded as a significant interaction.

## Results

**Fig. 2:**
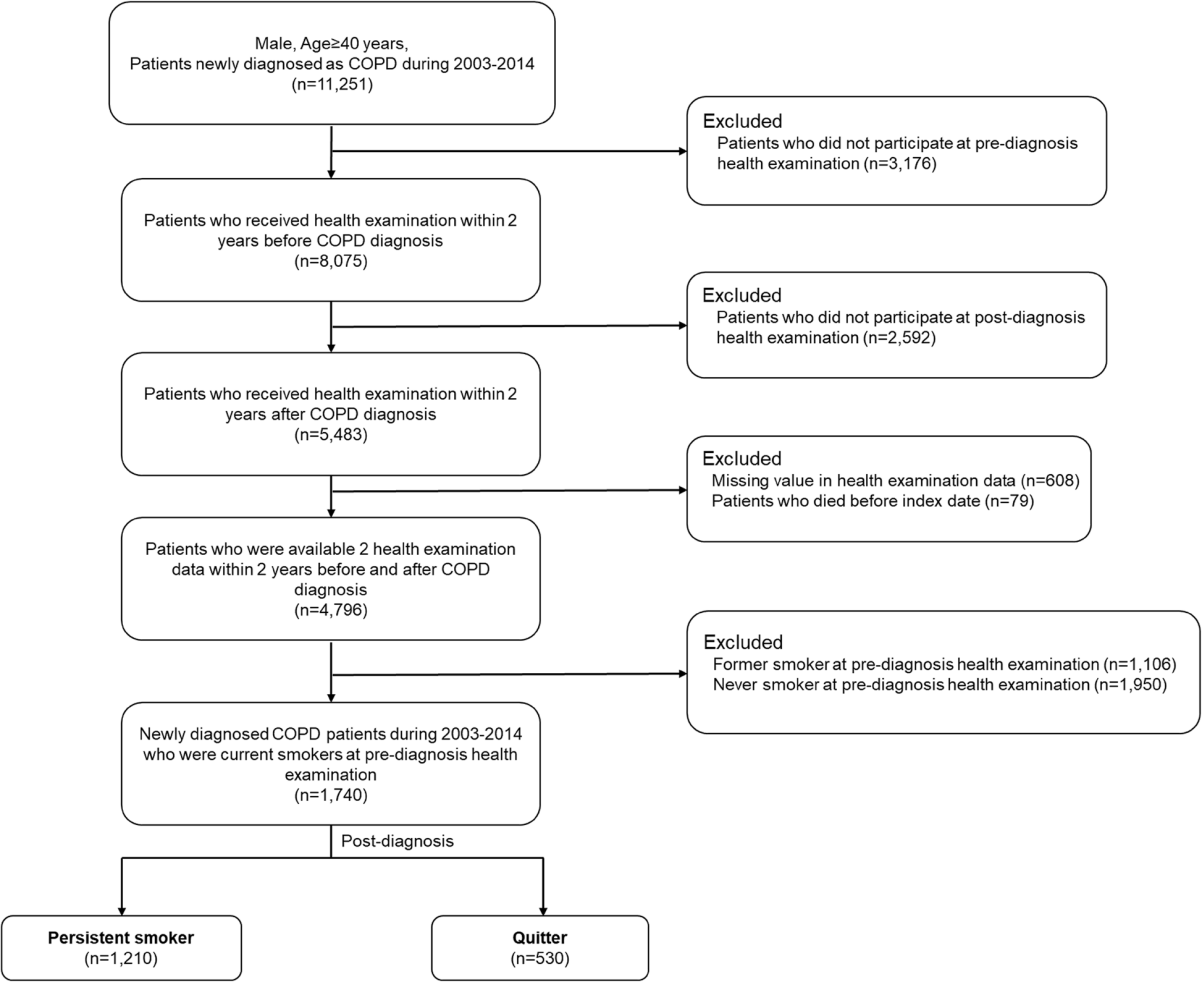
Flow diagram of selection of study subjects

Among 11,251 male patients aged 40 years or more who had been newly diagnosed with COPD between 2003 and 2014, 3,176 and 2,592 who did not undergo pre-diagnosis or post-diagnosis health examination, respectively, were excluded. Then, patients who had died (*n* = 79) before the index date or for whom there were missing covariates (*n* = 608) also were excluded. Finally, patients who had self-reported as former smokers (*n* = 1,106) or never smokers (*n* = 1,950) at the pre-diagnosis health examination were excluded as well. In total, 1,740 male patients who smoked at the time of their pre-diagnosis health examination and were newly diagnosed as COPD between 2003 and 2014 were included in this study (Fig. 2). The mean time span (standard deviation) between the first health examination and COPD diagnosis, between COPD diagnosis and the second health examination, and between the first and second health examination were 0.8(0.5), 0.9(0.6), and 1.8(0.6) years, respectively.

Among these 1,740 patients, 1,210 (69.5%) continued smoking after diagnosis of COPD, while 530 (30.5%) quit smoking. The baseline characteristics of the participants are presented in Table 1. Compared with persistent smokers, quitters were more likely to be older, drink alcohol less frequently, have higher systolic blood pressure, severe COPD, higher Charlson comorbidity index, and higher prevalence of cancer.

**Table 1:** Baseline characteristics of study population

	**Total**	**Persistent smoker**	**Quitter**	***p*-value**
**Number of participants**	1,740	1,210 (69.5%)	530 (30.5%)	
**Age, year, mean (SD)**	64.6 (9.0)	63.8 (8.9)	66.7 (8.8)	** < 0.0001**
< 65 years, N (%)	883 (50.8%)	663 (54.8%)	220 (41.5%)	** < 0.0001**
≥ 65 years, N (%)	857 (49.2%)	547 (45.2%)	310 (58.5%)	
**Household income, quartile, N (%)**				0.5891
1st (highest)	458 (26.3%)	324 (26.8%)	134 (25.3%)	
2nd	542 (31.2%)	371 (30.7%)	171 (32.3%)	
3rd	431 (24.8%)	307 (25.4%)	124 (23.4%)	
4th (lowest)	309 (17.7%)	208 (17.1%)	101 (19.0%)	
**Alcohol consumption, times per week, N (%)**				** < 0.0001**
0	750 (43.1%)	455 (37.6%)	295 (55.7%)	
0–1	326 (18.8%)	231 (19.1%)	95 (17.9%)	
1–2	243 (14.0%)	193 (16.0%)	50 (9.4%)	
3–4	223 (12.8%)	176 (14.6%)	47 (8.9%)	
≥ 5	198 (11.3%)	155 (12.7%)	43 (8.1%)	
**Physical exercise, times per week, N (%)**				0.3817
0	972 (55.9%)	665 (55.0%)	307 (57.9%)	
1–2	448 (25.8%)	313 (25.9%)	135 (25.5%)	
≥ 3	320 (18.3%)	232 (19.1%)	88 (16.6%)	
**Body mass index (BMI), kg/m**^**2**^**, mean (SD)**	22.9 (3.3)	22.9 (3.2)	23.0 (3.4)	0.5408
**Systolic blood pressure, mmHG, mean (SD)**	126.4 (16.0)	125.8 (15.9)	127.7 (16.2)	**0.0285**
**Total cholesterol, mg/dL, mean (SD)**	191.7 (38.9)	192.3 (38.6)	190.5 (39.4)	0.3643
**Fasting serum glucose, mg/dL, mean (SD)**	101.9 (26.8)	102.5 (28.2)	100.5 (23.2)	0.1295
**Charlson comorbidity index, mean (SD)**	3.9 (2.5)	3.8 (2.4)	4.2 (2.6)	**0.0040**
**COPD severity**				**0.0003**
Not severe	1,410 (81.0%)	1,008 (83.3%)	410 (77.4%)	
Severe	330 (19.0%)	202 (16.7%)	120 (22.6%)	
**Hypertension**				0.8281
No	1,355 (77.9%)	944 (78.0%)	402 (75.9%)	
Yes	385 (22.1%)	266 (22.0%)	128 (24.1%)	
**Cardiovascular disease**				0.2563
No	1,577 (90.6%)	1,103 (91.2%)	474 (89.4%)	
Yes	163 (9.4%)	107 (8.8%)	56 (10.6%)	
**Cancer**				**0.0275**
No	1,615 (92.8%)	1,134 (93.7%)	481 (90.8%)	
Yes	125 (7.2%)	76 (6.3%)	49 (9.2%)	

The chi-squared test was used to calculate the *p* values for the categorical variables, and analysis of variance (ANOVA) was used for continuous variables to compare baseline characteristics according to smoking status

During a mean follow-up duration of 7.6 years (standard deviation: 3.8 years), 564 deaths occurred. Table 2 shows that those who quit smoking after COPD diagnosis had a lower risk of all-cause (aHR, 0.83; 95% CI, 0.69–1.00) and cardiovascular (aHR, 0.56; 95% CI, 0.33–0.95) mortality relative to persistent smokers.

**Table 2:** Association between smoking status and risks of all-cause, cause-specific mortality among newly diagnosed COPD patients

Group	**Events**	**Person-years**	**Incidence Rate**	**Unadjusted HR (95% CI)**	**Adjusted HR (95% CI)**	***p*-value**
**All-cause mortality**						**0.049**
Persistent smoker	379	9387	404	1 (Reference)	1 (Reference)	
Quitter	185	3975	465	1.16 (0.97–1.38)	**0.83 (0.69–1.00)**	
**Cancer mortality**						
Persistent smoker	148	9387	158	1 (Reference)	1 (Reference)	0.495
Quitter	77	3975	194	1.23 (0.94–1.62)	0.91 (0.68–1.21)	
**Lung cancer mortality**						
Persistent smoker	88	9387	94	1 (Reference)	1 (Reference)	0.998
Quitter	49	3975	123	1.32 (0.93–1.87)	1.00 (0.69–1.45)	
**Cardiovascular mortality**						
Persistent smoker	60	9387	64	1 (Reference)	1 (Reference)	**0.030**
Quitter	20	3975	50	0.79 (0.48–1.31)	**0.56 (0.33–0.95)**	
**Ischemic heart disease mortality**						
Persistent smoker	15	9387	16	1 (Reference)	1 (Reference)	0.068
Quitter	4	3975	10	0.63 (0.21–1.89)	0.34 (0.11–1.07)	
**Stroke mortality**						
Persistent smoker	24	9387	26	1 (Reference)	1 (Reference)	0.586
Quitter	10	3975	15	0.99 (0.47–2.07)	0.82 (0.38–1.76)	
**Respiratory mortality**						
Persistent smoker	83	9387	88	1 (Reference)	1 (Reference)	0.449
Quitter	44	3975	110	1.26 (0.88–1.82)	0.86 (0.59–1.27)	
**COPD mortality**						
Persistent smoker	51	9387	54	1 (Reference)	1 (Reference)	0.060
Quitter	18	3975	45	0.84 (0.49–1.44)	0.58 (0.33–1.02)	

*Abbreviation*: *HR* hazard ratio

The incidence rate is per 10,000 person-years. The adjusted HR was calculated by Cox proportional hazards regression analysis after adjustment for age, household income, alcohol consumption, physical exercise, BMI, systolic blood pressure, total cholesterol, fasting serum glucose, Charlson comorbidity index, and COPD severity

Table 3 presents the results of the stratified analysis examining the relationship between smoking cessation and all-cause mortality among the subgroups, based on age, alcohol consumption, Charlson comorbidity index, severity of COPD, presence of hypertension, cancer, and cardiovascular disease. Although most of the results were not statistically significant, the overall findings were in line with the main results. Patients without cardiovascular disease who quit smoking after their COPD diagnosis had a lower risk of all-cause mortality (aHR, 0.80; 95% CI, 0.66–0.98). There was no significant interaction between the effect of smoking cessation and the risk of all-cause mortality for any of the above-listed variables.

**Table 3:** Stratified analysis of association between smoking status and all-cause mortality according to age, alcohol consumption, CCI, COPD severity, hypertension, cancer, and cardiovascular disease

Subgroup	**Persistent smoker**			**Quitter**			***p*-for interaction**
**Age group**^**a**^	Number	Events	Adjusted HR (95% CI)	Number	Events	Adjusted HR (95% CI)	
Age < 65 years	663	105	1 (Reference)	220	34	0.89 (0.60–1.33)	0.792
Age ≥ 65 years	547	274	1 (Reference)	310	151	0.91 (0.74–1.12)	
**Alcohol consumption**^**b**^							
No	455	295	1 (Reference)	170	124	0.83 (0.65–1.06)	0.652
Yes	755	235	1 (Reference)	209	61	0.80 (0.59–1.07)	
**CCI**^**c**^							
CCI score < 3	442	133	1 (Reference)	170	49	0.78 (0.56–1.10)	0.557
CCI score ≥ 3	768	246	1 (Reference)	360	136	0.88 (0.71–1.10)	
**COPD severity**^**d**^							
Not severe COPD	1,008	316	1 (Reference)	402	63	0.86 (0.70–1.06)	0.42
Severe COPD	202	144	1 (Reference)	128	41	0.78 (0.50–1.21)	
**Hypertension**^e^							
No	944	234	1 (Reference)	411	116	0.85(0.67–1.08)	0.799
Yes	266	145	1 (Reference)	119	69	0.92(0.67–1.25)	
**Cancer**^e^							
No	1,134	340	1 (Reference)	481	163	0.84(0.69–1.02)	0.777
Yes	76	39	1 (Reference)	49	22	0.76(0.42–1.38)	
**Cardiovascular disease**^e^							
No	1,103	339	1 (Reference)	474	158	0.80 (0.66–0.98)	0.567
Yes	107	40	1 (Reference)	56	27	1.24 (0.69–2.22)	

*Abbreviations*: *HR* hazard ratio, *CI* confidence interval, *CCI* Charlson comorbidity index

^**a**^Values are represented as adjusted HR (95% CI) after adjusting for confounding factors (household income, alcohol consumption, physical exercise, BMI, systolic blood pressure, total cholesterol, fasting serum glucose, CCI, and COPD severity) other than age

^**b**^All confounding factors except for alcohol consumption using multivariate Cox proportional regression model

^**c**^All confounding factors except for CCI using multivariate Cox proportional regression model

^**d**^All confounding factors except for COPD severity using multivariate Cox proportional regression model

^e^All confounding factors (age, household income, alcohol consumption, physical exercise, BMI, systolic blood pressure, total cholesterol, fasting serum glucose, CCI, and COPD severity) using multivariate Cox proportional regression model

The supplementary analysis revealed that persistent smokers had a higher risk of all-cause mortality (aHR, 95% CI;1.20, 1.00–1.44) compared with quitters, with a population-attributable fraction of 11.4% and a “number needed to treat at ten-year follow up” of 21.0.

## Discussion

In this retrospective cohort study using a nationwide database, we showed that subjects who had quit relatively soon (i.e., within two years) after COPD diagnosis had significantly lower all-cause and cardiovascular mortality risks relative to persistent smokers. The relative risk reduction for quitting smoking for all-cause and cardiovascular mortality was approximately 17 and 44%, respectively. The risk-reducing impact of quitting smoking did not vary significantly based on age, alcohol consumption, Charlson comorbidity index, severity of COPD, presence of hypertension, cancer or cardiovascular disease.

COPD diagnosis is significant, as it can serve as a wake-up call about the negative impacts of smoking on respiratory health [7]. Early implementation of smoking cessation is highly important, as it can help to slow the progression of the disease [20] and reduce the risk of complications such as cardiovascular disease [21]. Our data showed that a significant number of patients who had recently been diagnosed with COPD continued to smoke afterwards (69.5%). In light of the fact that 11.4% of the COPD deaths in our study were attributed to persistent smoking, improving the cessation rate of COPD sufferers may contribute to prevention of future deaths. Our study also indicated that successful quitting by 21 persistent smokers may result in the prevention of one case of death within 10 years. The results of our study could be used to support the current recommendation of smoking cessation and to remind smokers of the benefits of quitting smoking as near to the time of diagnosis as possible.

Two previous studies have investigated the effect of smoking status at a specific point in time on the outcome of COPD and found that ex-smokers had a higher survival rate than persistent smokers [8, 22]. However, these studies could not determine whether smoking cessation after COPD diagnosis affects mortality. Three other studies have compared smoking status at two points in time, but included only an analysis of the post-COPD diagnosis period [9–11]. One of these, a clinical trial, convincingly showed that quitters had lower all-cause mortality compared with persistent smokers, but it had considered only young, asymptomatic COPD patients^11^. The other two studies showed similar results, but had included only a small number of patients with a short follow-up duration [9, 10]. In contrast to the previous studies, our study included newly diagnosed male COPD patients regardless of severity and investigated how smoking cessation affects mortality from various diseases, all of which are common causes of death in COPD patients.

The benefits of quitting smoking after COPD diagnosis appear to be similar to those for the general population in terms of lowering all-cause and cardiovascular mortality. Previous study on Asian populations have found that all-cause and cardiovascular mortality decreased in a dose–response manner with time since quitting [23]. Quitting smoking may not completely reverse impaired lung function in COPD patients [5], but it can still prevent, or at least delay, future deaths. Notwithstanding, the risk of cancer mortality was not significantly reduced in quitters relative to persistent smokers in the present study. A previous study involving mild-to-moderate COPD patients without symptoms found, consistently with the current results, that the relationship between quitting smoking and cancer mortality was uncertain within the first decade after smoking cessation [11]. To accurately determine the link between quitting smoking and cancer mortality in newly diagnosed COPD patients, a longer follow up will be necessary.

Multiple mechanisms may contribute to the increased mortality in COPD patients due to smoking. Smoking and inhalation exposure can increase the production of certain growth factors and lead to remodeling in the airway epithelium [24]. Also, smoking can deplete the levels of intraluminal secretory IgA, leading to macrophage accumulation and resultant peripheral lung inflammation [25, 26]. It is suggested that peripheral lung inflammation induces “spill-over” of cytokines into systemic circulation [27] and worsens comorbid disease [26]. Smoking also causes vasodilatory dysfunction, increased thrombogenicity, and elevated levels of low-density lipoprotein [28–30]. After smoking cessation, coronary artery vasomotor function and airway hyperresponsiveness are improved [31, 32] and bronchial epithelial remodeling is reversed to some extent [33]. Smoking cessation, by alleviation of the acute inflammatory process, also can lead to reduced risk of COPD exacerbation [34] and improved bronchodilator response [35].

Our study has several limitations. First, a self-reported questionnaire was used to determine smoking status, which may not be completely reliable. Future studies should use more accurate methods such as urine cotinine or carboxyhemoglobin tests. Second, due to the limited number of participants who had undergone health examinations after the index date, we did not account for any further changes in smoking behavior after the second health examination. Since it is common for people who have quit smoking to start smoking again [20], this could have caused underestimation of the beneficial effect of smoking cessation. Third, COPD severity was not based on the extent of restricted airflow. We attempted to compensate for this limitation by defining severe COPD based on the use of medication to treat COPD exacerbation. Fourth, due to missing data, our study did not consider the number of pack-years, which is the total amount of smoking exposure a person has experienced over time. Fifth, the findings of our study are limited to male patients and cannot be generalized to female patients. Finally, the number of cases where both pre- and post-diagnosis health checkups are conducted has decreased by over 50% as a result of many individuals not receiving health checkups twice in succession. ur study also has several strengths. First, it was conducted on a large, nationwide population in Korea. Second, we compared two time points before and after COPD diagnosis to determine the benefits of quitting smoking after being diagnosed with COPD. Third, our analysis considered a wide range of confounding factors in terms of sociodemographic variables. Fourth, the study participants were completely followed up from the index date until their date of death or until the end of the cohort period.

## Conclusion

Quitting smoking within 2 years after COPD diagnosis was associated with lower risk of all-cause and cardiovascular mortality relative to persistent smokers. The results of our study suggest that quitting smoking after COPD diagnosis can lead to significant health benefits, and as such, support the current recommendation of smoking cessation.

## Supplementary Information

**Additional file1: **Supplementary Table 1.

## Data Availability

The data used to support the conclusions in this piece is not publicly available. Researchers interested in accessing the data should contact the Korean National Health Insurance Service (NHIS). Due to ethical guidelines set by the Korean NHIS, the datasets used in the research cannot be shared. However, researchers can still obtain the raw datasets by submitting a proposal on the Korean NHIS website (https://nhiss.nhis.or.kr).

## Abbreviations

aHR: Adjusted hazard ratio
CI: Confidence interval
