# Supplementary material for: Smoking cessation after diagnosis of COPD is associated with lower all-cause and cause-specific mortality: a nationwide population-based cohort study of South Korean men
Source: BMC Pulm Med. 2023 Jul 3;23:237. doi: 10.1186/s12890-023-02533-1 (PMC10316560; doi:10.1186/s12890-023-02533-1)
Supplement: Supplementary file 1 — Additional file1: Supplementary Table 1. [file 12890_2023_2533_MOESM1_ESM.docx]

**Supplementary Table 1.**

Estimated hazard ratio of all-cause mortality among newly diagnosed COPD patients using Cox proportional hazards model.

|  | | **Persistent smoker** | **Quitter** |
| --- | --- | --- | --- |
| **All-cause mortality** | |  |  |
|  | **Cases, n** | 379 | 185 |
|  | **Person-years** | 9387 | 3975 |
|  | **aHR^*^(95% CI)** | 1.20 (1.00-1.44) | 1(Reference) |
|  | ***p*-value** | 0.049 |  |
|  | **Population attributable fraction^**^,%** | 11.4% |  |
|  | **NNT^†^ at 10-year follow-up** | 21.0 |  |

Abbreviations aHR, adjusted hazard ratio; CI, confidence interval; NNT, numbers needed to treat;

**^*^**Adjusted hazard ratio calculated by cox proportional hazards regression analysis after adjustment for age, household income, alcohol consumption, physical exercise, BMI, systolic blood pressure, fasting serum glucose, total cholesterol, Charlson comorbidity index, and COPD severity.

**^**^**calculated by p*(1-(1/HR)) where p is the prevalence of persistent smokers among outcome cases.

**^†^**calculated by 1/([S_c_(t)]^h^-Sc(t)) where S_c_(t) is the survival probability of the persistent smoker group and h is the hazard ratio of the persistent smoker group referent to the quitter group.
